# Supplementary material for: Biocontrol Potential of Bacillus stercoris Strain DXQ-1 Against Rice Blast Fungus Guy11
Source: Microorganisms. 2025 Jun 30;13(7):1538. doi: 10.3390/microorganisms13071538 (PMC12300960; doi:10.3390/microorganisms13071538)
Supplement: Supplementary file 1 [file microorganisms-13-01538-s001.zip › Supplementary Tables S1-S3.pdf]

Supplementary Table S1. **Inhibition rate of candidate biocontrol strains against *M. oryzae* GUY11.** The inhibition rate (%) was calculated based on the colony diameter reduction on PDA plates compared to the control. Data represent the mean ± SD of three replicates.

Candidate biocontrol strain	Inhibition rate against *M. oryzae* GUY11 (%) ± SD	Inhibition Level
XQ2	39.54±0.56	Strong
87	39.54±0.34	Strong
106	9.30±0.22	Moderate
⑦	39.54±0.88	Strong
⑥	38.86±0.96	Strong
96	10.31±0.35	Moderate
82	12.66±0.47	Moderate
DXQ-1	40.20±0.34	Strong

Supplementary Table S2.\$**Effects of DXQ-1 fermentation broth on spore germination and appressorium formation of *M. oryzae*.** Spore germination rate and appressorium formation rate were assessed microscopically at 4 h, 8 h, 24 h post-inoculation. Data represent mean ± SD from three independent experiments. Asterisks indicate significant differences compared to the control (Student's t-test, *$p < 0.05$).

Dilution ratio	CK		100%		10%		1%	
Germination time	Germination rate	Appressorium formation rate (%) ± SD	Germination rate (%) ± SD	Appressorium formation rate (%) ± SD	Germination rate (%) ± SD	Appressorium formation rate (%) ± SD	Germination rate (%) ± SD	Appressorium formation rate (%) ± SD
4h	100.00 ± 0.19	44.44 ± 0.21	1.54 ± 0.12	0.00	1.92 ± 0.33	0.00	37.10 ± 0.30	34.78 ± 0.40
8h	100.00 ± 0.16	45.57 ± 0.28	64.41 ± 0.18	0.00	92.00 ± 0.36	0.00	100.00	43.33 ± 0.31
24h	100.00 ± 0.13	100.00 ± 0.17	83.33 ± 0.11	0.00	100.00 ± 0.29	6.25 ± 0.25	100.00	98.75 ± 0.29

Supplementary Table S3. Plant height, fresh weight, and dry weight of TP309 rice plants after inoculation with DXQ-1 fermentation broth.

	Plant height (cm)	Fresh weight (g)	Dry weight (g)
CK	37.70 ± 0.84	0.15 ± 0.02	0.03 ± 0.01
DXQ-1	44.37 ± 2.56*	0.21 ± 0.07*	0.04 ± 0.01*

* indicated $p \leq 0.05$
