# Supplementary figures and images for: Biocontrol Potential of Bacillus stercoris Strain DXQ-1 Against Rice Blast Fungus Guy11
Source: Microorganisms. 2025 Jun 30;13(7):1538. doi: 10.3390/microorganisms13071538 (PMC12300960; doi:10.3390/microorganisms13071538)

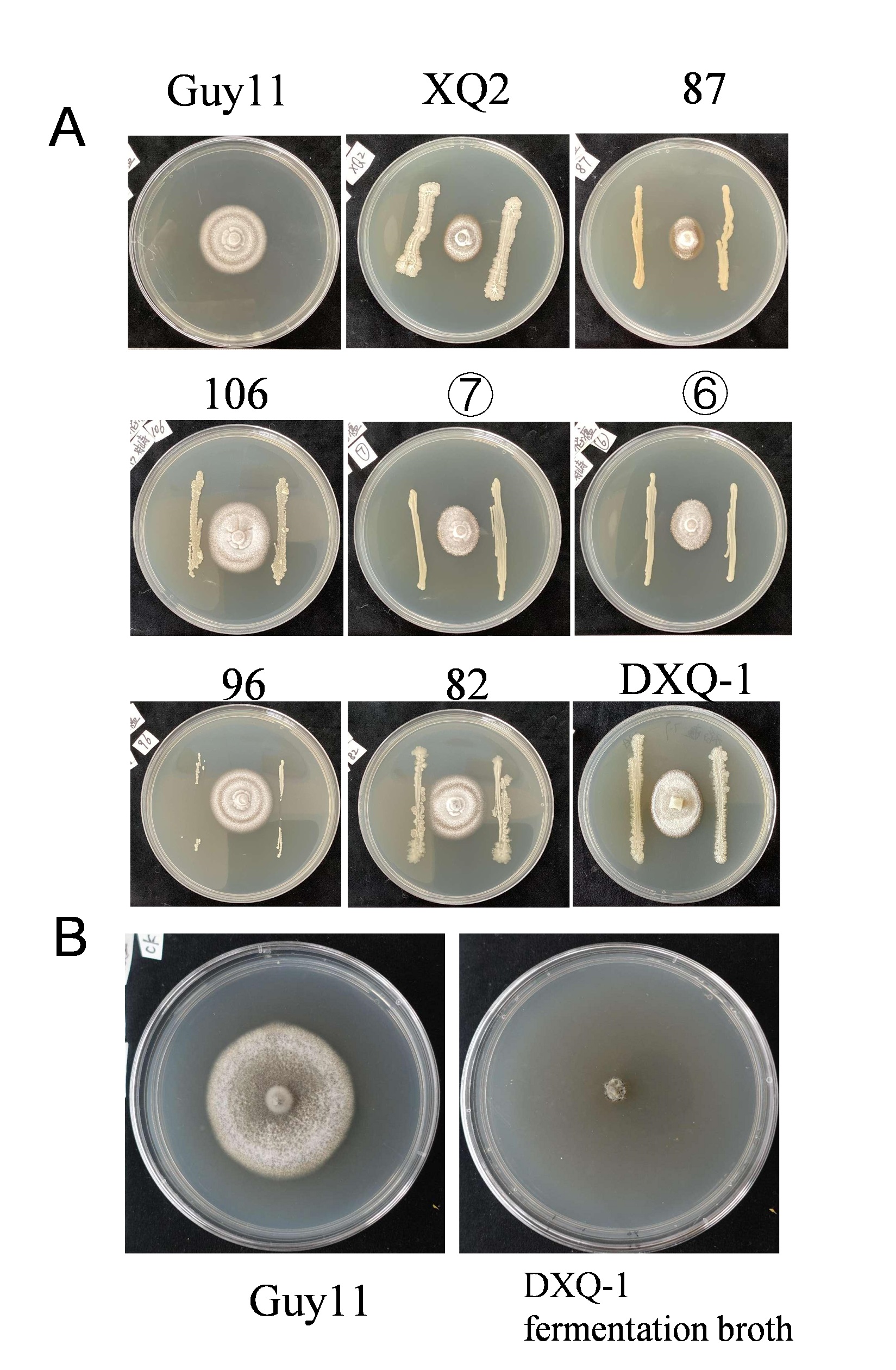

Supplement: Supplementary file 1 [file microorganisms-13-01538-s001.zip › Figure S1.jpg]

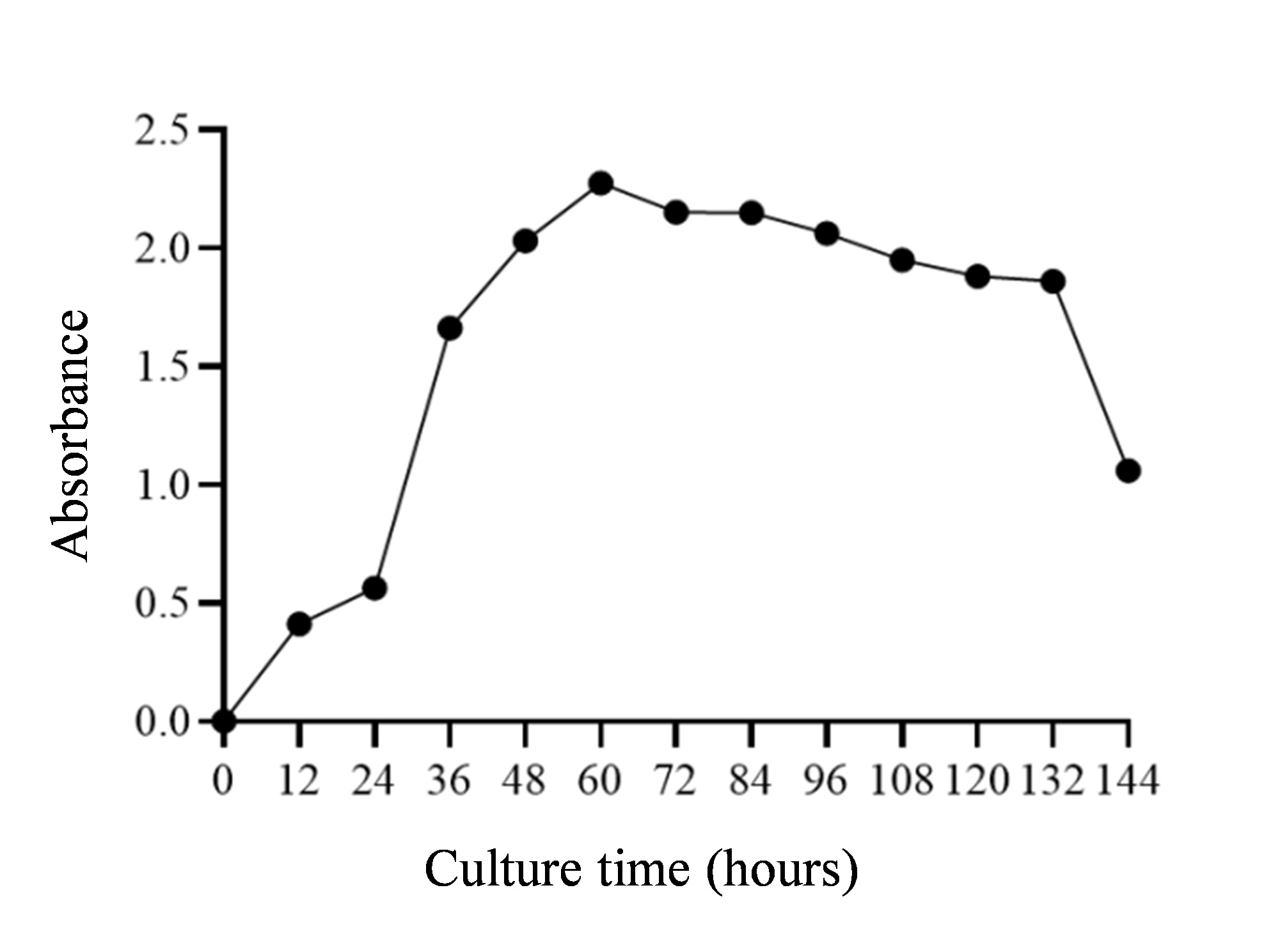

Supplement: Supplementary file 1 [file microorganisms-13-01538-s001.zip › Figure S2.jpg]

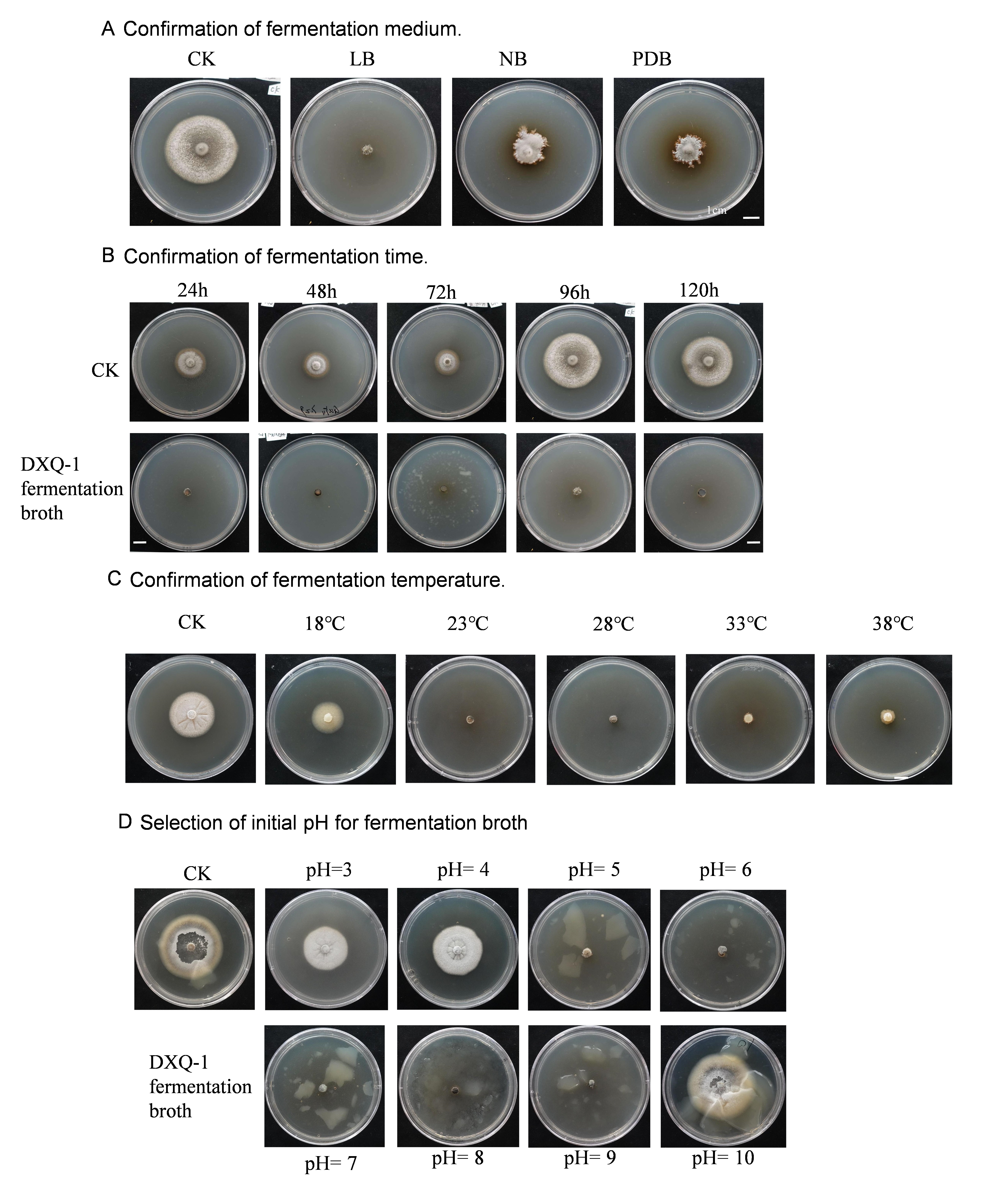

Supplement: Supplementary file 1 [file microorganisms-13-01538-s001.zip › Figure S3.jpg]
